# iASPP overexpression is associated with clinical outcome in spinal chordoma and influences cellular proliferation, invasion, and sensitivity to cisplatin *in vitro*

**DOI:** 10.18632/oncotarget.20190

**Published:** 2017-08-11

**Authors:** Yunlong Ma, Bin Zhu, Xiaoguang Liu, Zhongjun Liu, Liang Jiang, Feng Wei, Miao Yu, Fengliang Wu, Hua Zhou, Nanfang Xu, Xiao Liu, Lei Yong, Yongqiang Wang, Peng Wang, Chen Liang, Guanping He

**Affiliations:** ^1^ Department of Orthopedics, Peking University Third Hospital, Beijing 100191, China; ^2^ The Center for Pain Medicine, Peking University Third Hospital, Beijing 100191, China

**Keywords:** chordoma, iASPP, prognosis, invasion, cisplatin

## Abstract

The oncogenetic function of inhibitory member of the apoptosis stimulating protein of p53 family (iASPP) in chordoma is unclear and remains to elucidate. The expression of iASPP in chordoma tissues and cells, its correlation to clinicopathological parameters and the effect on the patients’ prognosis were evaluated. Cellular proliferation, invasion and cisplatin-response were observed after the iASPP knockdown or overexpression *in vitro*. Co-Immunoprecipitation assay was used to explore the interaction between iASPP and p53. The regulation of miRNA-124 on the expression and apoptotic function of iASPP was explored after transiently transfecting cells with miRNA-124 mimics or inhibitor. Results indicated that iASPP overexpressed in chordoma tissues and cells. Its overexpression was associated with tumor invasion and local recurrence, and was predictive of patients’ poor prognosis. Cells with iASPP-silence showed a decreased ability of proliferation and invasion, but an increasing sensitivity to cisplatin. Besides, iASPP could combine with p53 in either endogenous or exogenous detection. Post-transcriptionally, miRNA-124 negatively regulated the expression of iASPP, which further led to the changes of apoptosis-related proteins. Thus, iASPP overexpression is associated with the clinical outcome in spinal chordoma and influences cellular proliferation, invasion, and the sensitivity to cisplatin.

## INTRODUCTION

Chordoma is a primary malignant bone tumor originated from primitive notochord remnants with strong local invasiveness and a high recurrence rate. It accounts for 1∼4% of all primary malignant skeletal tumors and the overall incidence is relatively low [1, 2]. The 5- and 10-year survival rates of the patients are approximately 68% and 40%, respectively [3]. Standard treatment of chordoma consists of surgery and radiotherapy due to its resistance to chemotherapeutics [4, 5]. Even the patients received extensive surgical resection, local recurrence is still common [6, 7]. Although the excellent response to radiotherapy reported, the clinical application and treatment efficacy of this adjuvant therapy are still limited and debated [8, 9]. Given this, the identification of novel biomarkers could be helpful for the prediction, diagnosis and precise target therapy for chordoma.

The dysfunction of p53 is an important process in tumorigenesis, and mainly results from p53 mutation or inactivation [10]. Although p53 dysfunction is associated with chordoma tumorigenesis [11, 12], genetic alternation of the encoding gene appears to be an uncommon event as mutations in specific exons of p53 have not been observed [11]. In addition, although the loss of heterozygosity on chromosome 17p13, where p53 located, has been reported in some chordoma cases, this event is not correlated to abnormal p53 expression or overall survival rate [12]. These findings suggest that mutation may be not the primary cause of p53 dysfunction in chordoma.

iASPP, the inhibitory member of the apoptosis stimulating protein of p53 (ASPP) family, is encoded by *PPP1R13L* gene [13]. It was initially identified as Rel-associated inhibitor (RAI), as its interaction with nuclear factor kappa B (NF-κB) subunit p65 (RelA) suppresses its transcriptional activity [14]. iASPP is the exclusive, evolutionarily conserved p53 suppressor in this family, whereas another two members, namely ASPP1 and ASPP2 are p53 activators [14, 15]. iASPP has been reported to overexpress in many human tumors, including non-small cell lung cancer, breast cancer, hepatocellular carcinoma, cervical cancer, and leukemia [16–20]. Its overexpression confers proliferative, migratory, and invasive capacity to cancer cells, and is associated with tumor recurrence, chemotherapy tolerance, and poor prognosis [16, 19, 21–23]. Inhibition of iASPP expression or disruption of iASPP-p53 interaction in tumor cells induces apoptosis and growth suppression in tumor cells, and even enhances chemotherapy sensitivity [24, 25]. Recently, growing studies have reported the cancer-related roles of microRNAs (miRNAs) [26, 27] and their regulation on iASPP expression [28, 29]. Altered expression of iASPP in many tumor cells can be post-transcriptionally regulated by various miRNAs such as miRNA-124 [30–33], miRNA-140 [34, 35], miRNA-184 [36] and miRNA-506 [37], which involve in modulating the growth or invasion of these cells by targeting iASPP. Therein, studies have well documented that the 3′-UTR of iASPP includes the binding sites of miRNA-124, indicating the important role of miRNA-124 in the regulation of iASPP.

To our knowledge, no study has thus far reported the function of iASPP in chordoma. In view of the strong local invasion and chemotherapy tolerance of chordoma as well as the important oncogenetic function of iASPP reported in other tumors, iASPP expression in chordoma and its exact regulation on the cellular proliferation, invasiveness, and cisplatin response were observed in this study. What's more, the potential regulation of miRNA-124 on apoptosis function of iASPP was also explored.

## RESULTS

### Characterization of tumor tissues as conventional-type chordomas

The results of hematoxylin and eosin (HE) staining showed that all samples presented a classic lobular architecture separated by fibrous septa. Sheets or elongated cords of clear cells with multiple intracytoplasmic vacuoles distributed in tumor nests with abundant eosinophilic cytoplasm and round nuclei within myxoid matrices, which is the pathognomonic feature of conventional-type chordoma (Supplementary Figure 1A). In addition, these samples were identified based on immunohistochemistry (IHC), staining for diagnostic biomarkers of chordoma including cytokeratin, vimentin, EMA, S-100, and brachyury (Supplementary Figure 1B–1F).

### iASPP is overexpressed in chordoma tissues and cells

**Figure 1:**
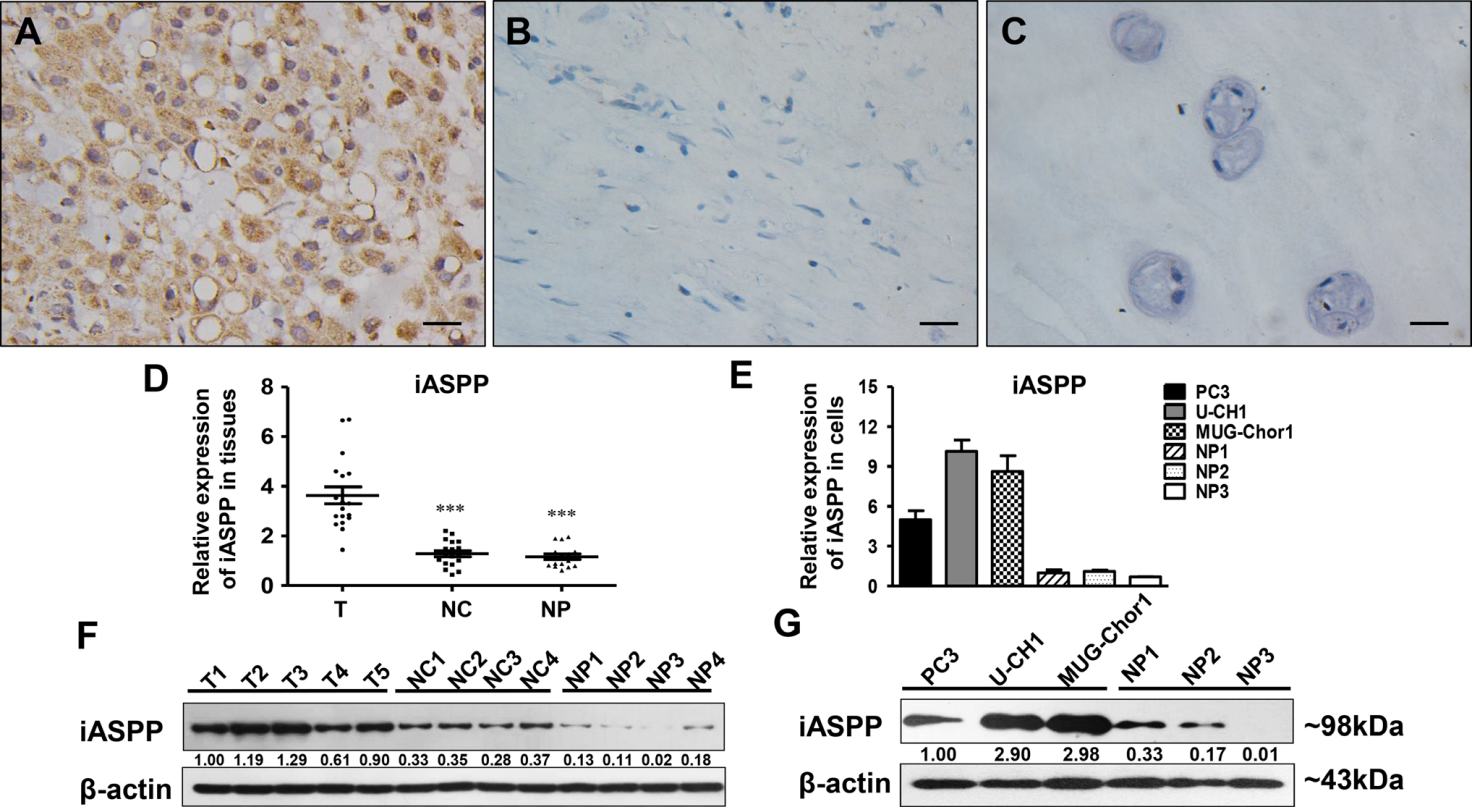
iASPP expression in chordoma tissues and cells (**A**–**C**) Immunohistochemistry staining of iASPP in chordoma tissues (A), distant normal control tissues (B), and nucleus pulposus (C). T: tumor group, NC: normal control tissues group, NP: nucleus pulposus tissues group. (**D**) Relative expression of iASPP in tissues as detected by RT-qPCR. ^**^*p < 0.001 *vs*. tumor group. (**E**) Relative expression of iASPP in cells as detected by RT-qPCR. (**F**) Relative expression of iASPP in tissues as detected by WB. (**G**) Relative expression of iASPP in cells as detected by WB. Numbers below pictures of F and G represent semi-quantitative analysis of each line by Quantity One software. Bar scale = 20 μm.

IHC showed that iASPP predominantly expressed in the cytoplasm of chordoma cells with diverse intensities (Supplementary Figure 2A–2D). iASPP expression in chordoma tissues was elevated (Figure 1A) when compared to that in distant normal tissues (Figure 1B) and nucleus pulposus (NP) (Figure 1C) with a statistically significant difference (*p* = 0.000, iASPP expression in chordoma vs. distant normal tissues; *p* = 0.009, iASPP expression in chordoma vs. NP) (Table 1). Consistently, iASPP overexpression in chordoma tissues and cells was also confirmed by either real-time quantitative polymerase chain reaction (RT-qPCR) (Figure 1D, 1E) or western blotting (WB) (Figure 1F, 1G).

**Table 1:** Expression of iASPP in chordoma, distant normal tissues and nucleus pulposus samples

Tissue sample	*N*	iASPP expression		*p* value
		High (%)	Low (%)	
Chordoma tissues	31	21 (67.7 %)	10 (32.3 %)	
Distant normal tissue	18	2 (11.1 %)	16 (88.9 %)	0.000*
Nucleus pulposus	15	4 (26.7 %)	11 (73.3 %)	0.009*

### iASPP overexpression correlates to tissue invasion, tumor recurrence, and poor prognosis

**Figure 2:**
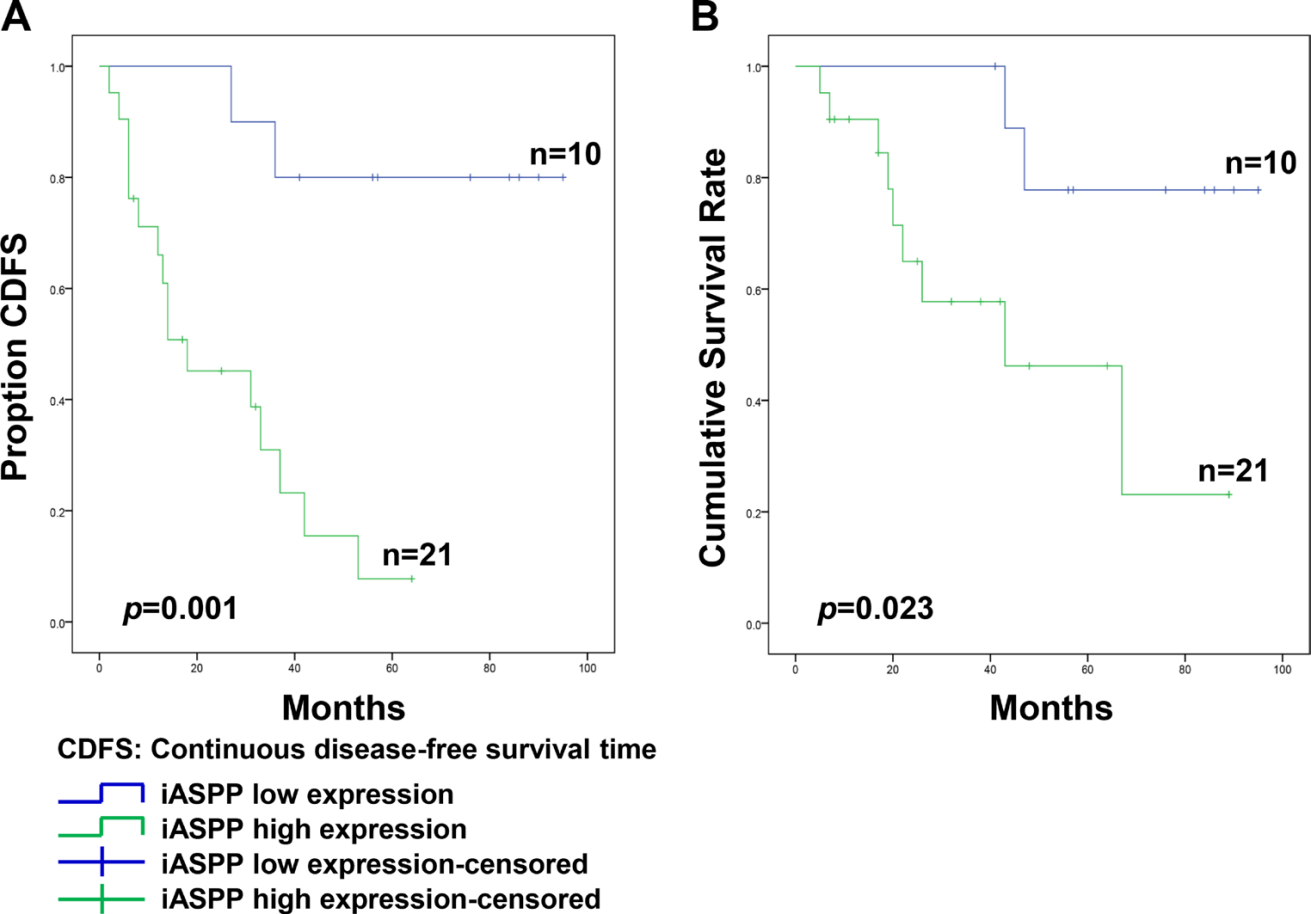
The association of iASPP expression with the prognosis of patients with spinal chordoma (**A** and **B**) Kaplan-Meier survival analysis and log-rank test of continuous disease-free survival (A) and cumulative survival rate (B).

Pearson's Chi-square analysis indicated that high iASPP expression was not statistically associated with patient age (*p* = 0.214), gender (*p* = 0.445), tumor location (*p* = 0.919), or tumor size (*p* = 0.935) (Table 2). However, samples from patients that presented surrounding tissue invasion exhibited increased iASPP positivity (88.9%, 16/18) compared to that in samples from patients without surrounding invasion (38.5%, 5/13), and this difference was statistically significant (*p* = 0.003; Table 2). During follow-up, 18 patients experienced local recurrence and the median recurrence time was 20.1 ± 14.8 months. Based on all samples with high iASPP expression, 16 of 21 patients (76.2%) developed recurrence, whereas in samples with low iASPP expression, only 2 of 10 patients (20.0%) experienced recurrence, and this difference was statistically significant (*p* = 0.003). At the end of follow-up, tumor-related death occurred in 11 patients, including two cases of low iASPP expression and nine cases of high iASPP expression. Furthermore, a Kaplan-Meier survival plot and a log-rank test also suggested that the continuous disease-free survival time (CDFS) of patients with high iASPP expression was much shorter than that in patients with low iASPP expression (*p* = 0.001; Figure 2A). Similarly, the cumulative total survival rate in the high expression group was notably lower than that in the low expression group (*p* = 0.023; Figure 2B).

**Table 2:** Association of iASPP expression with clinical parameters in conventional chordoma

Parameters	*N*	iASPP expression		*p* value
		High	Low	
Age (years)				
< 50	11	9	2	0.214
≥ 50	20	12	8	
Gender				
Male	22	14	8	0.445
Female	9	7	2	
Tumor Location				
Cervical	19	13	6	0.919
Sacrococcygeal	12	8	4	
Tumor size (mm)				
< 90	22	15	7	0.935
≥ 90	9	6	3	
Surrounding tissue invasion				
Yes	18	16	2	0.003*
No	13	5	8	
Recurrence				
Yes	18	16	2	0.003*
No	13	5	8	

### Cellular proliferation is decreased by iASPP knockdown but increased by iASPP overexpression

**Figure 3:**
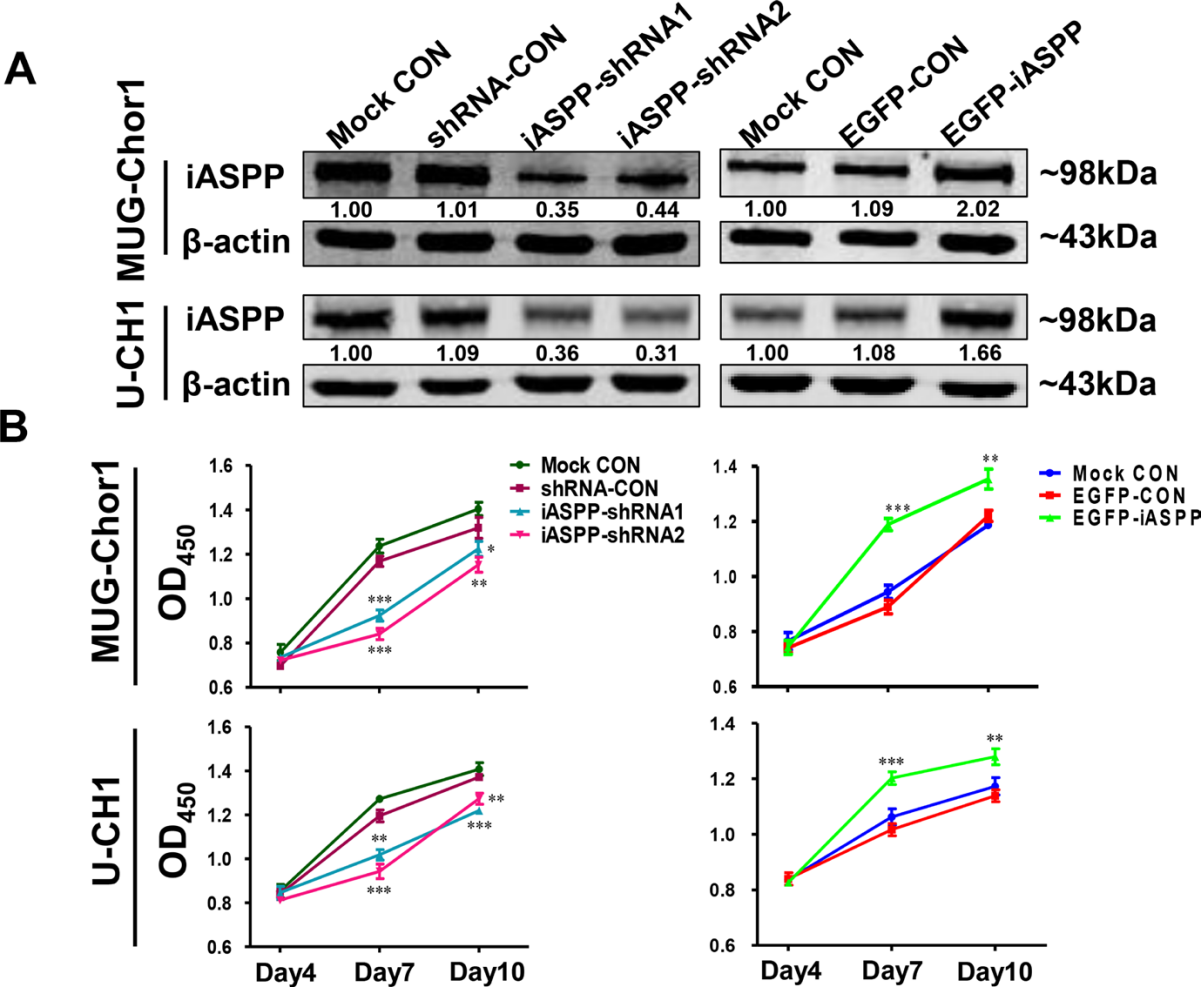
The extrinsic knockdown and overexpression of iASPP and their effects on cells proliferation based on CCK-8 assays (**A**) The extrinsic knockdown and overexpression of iASPP in MUG-Chor1 and U-CH1 cells as identified by WB. Numbers below pictures represent semi-quantitative analysis of each line by Quantity One software. (**B**) Proliferation of both cells with iASPP knockdown and overexpression. *n* = 3. Mean ± SEM. *p < 0.05, ^*^p < 0.01, ^**^*p < 0.001, iASPP-shRNA1 group or iASPP-shRNA2 group *vs*. shRNA-CON group, and EGFP-iASPP group *vs*. EGFP-CON group. CON: control.

In order to explore the potential function of iASPP gene in chordoma, we established the knockdown and overexpression systems of iASPP by transfecting both cells with pLKD-iASPP-shRNAs and pLenti-EGFP-iASPP. The results of WB assay confirmed that endogenous iASPP successfully silenced by pLKD-iASPP-shRNAs and overexpressed by pLenti-EGFP-iASPP (Figure 3A).

The effect of iASPP overexpression and knockdown on cellular proliferation was determined using CCK-8 and colony formation assays. CCK-8 assay (Figure 3B) demonstrated that when compared to that in the pLKD-shRNA-Control group, the proliferation of MUG-Chor1 cells harboring pLKD-iASPP-shRNA1 and pLKD-iASPP-shRNA2 was remarkably reduced after culturing for 4 d (0.79-fold, *p* < 0.001 and 0.72-fold, *p* < 0.001, respectively) and 7 d (0.94-fold, *p* < 0.05 and 0.87-fold, *p* < 0.01, respectively). However, the proliferation of MUG-Chor1 cells expressing pLenti-EGFP-iASPP was notably increased after culture for 4 d (1.34-fold, *p* < 0.001) and 7 d (1.11-fold, *p* < 0.01) when compared to that of the pLenti-EGFP-Control group. Similarly, the proliferation of U-CH1 cells with pLKD-iASPP-shRNA1 and pLKD-iASPP-shRNA2 also obviously reduced after culturing for 4 d (0.85-fold, *p* < 0.01 and 0.79-fold, *p* < 0.001, respectively) and 7 d (0.89-fold, *p* < 0.001 and 0.93-fold, *p* < 0.01, respectively). In contrast, the proliferation of U-CH1 cells harboring pLenti-EGFP-iASPP prominently increased after culturing for 4 d (1.18-fold, *p* < 0.001) and 7 d (1.12-fold, *p* < 0.01).

**Figure 4:**
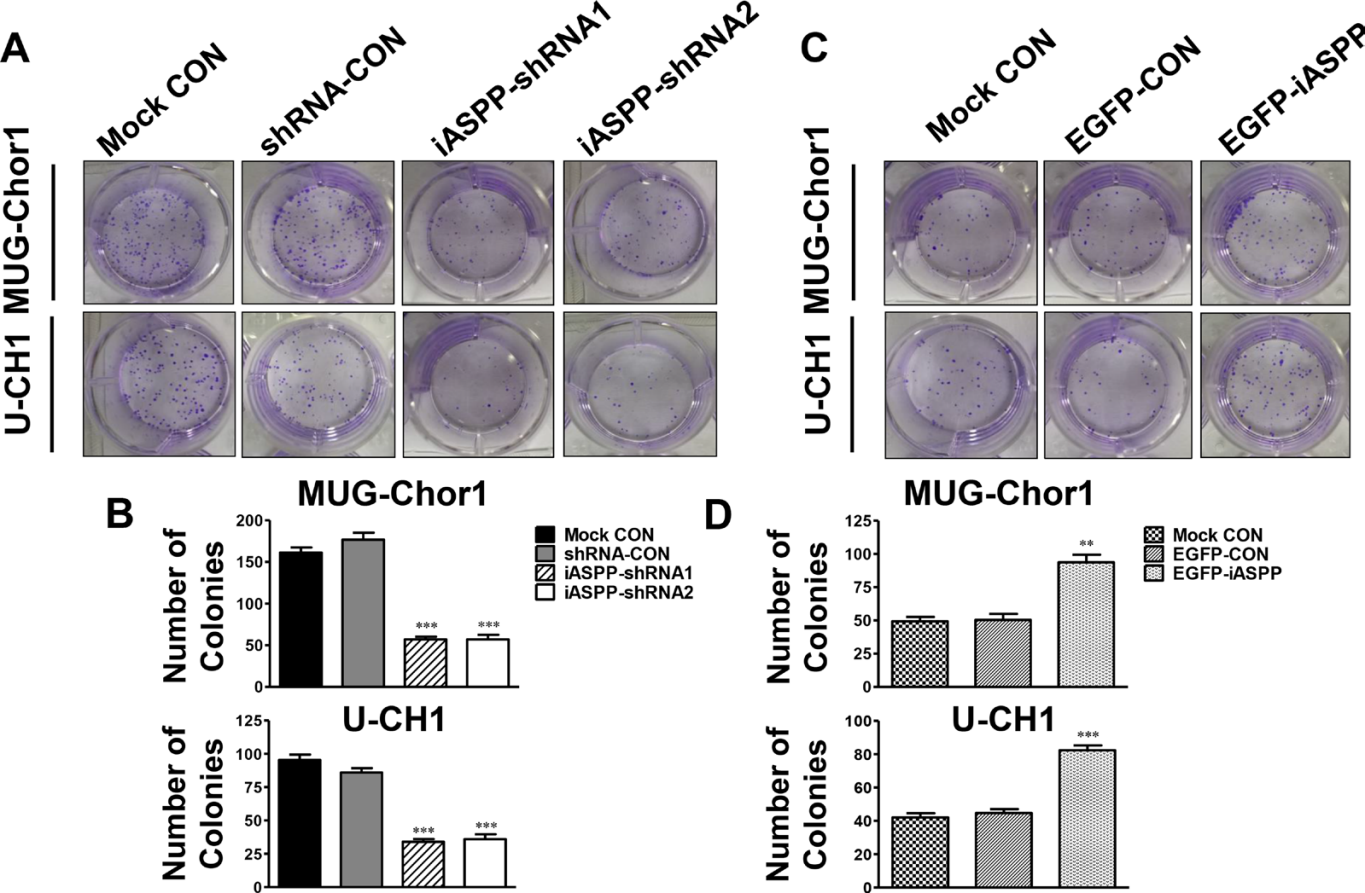
Effect of iASPP knockdown and overexpression on colony formation (**A**) Pictures of colony formation assay in MUG-Chor1 and U-CH1 cells after the iASPP knockdown with iASPP-shRNAs. (**B**) The number of colonies in both cells after the iASPP knockdown with iASPP-shRNAs. (**C**) Pictures of colony formation assay in MUG-Chor1 and U-CH1 cells after the iASPP overexpression with EGFP-iASPP. (**D**) The number of colonies in both cells after the iASPP overexpression with EGFP-iASPP. *n* = 3. Mean ± SEM. ^*^p < 0.01, ^**^*p < 0.001, iASPP-shRNA1 group or iASPP-shRNA2 group *vs*. shRNA-CON group, and EGFP-iASPP group *vs*. EGFP-CON group. CON: control.

Accordingly, colony formation in the two iASPP-silenced groups was lower than that in the corresponding control group (Figure 4A). The corresponding fold change was about 0.45-fold in the iASPP-shRNA1 group (*p* < 0.001) and 0.42-fold in the iASPP-shRNA2 (*p* < 0.001) for MUG-Chor1, and 0.54-fold in the iASPP-shRNA1 group (*p* < 0.001) and 0.59-fold in the iASPP-shRNA2 (*p* < 0.001) for U-CH1 (Figure 4B). However, the number of colonies formed in the iASPP-overexpressing group was dramatically higher than that in the corresponding control group (Figure 4C), which was approximately 2.00-fold for MUG-Chor1 (*p* < 0.001) and 1.97-fold for U-CH1 (*p* < 0.001) (Figure 4D).

### Cells invasion is suppressed by iASPP knockdown but induced by iASPP overexpression

**Figure 5:**
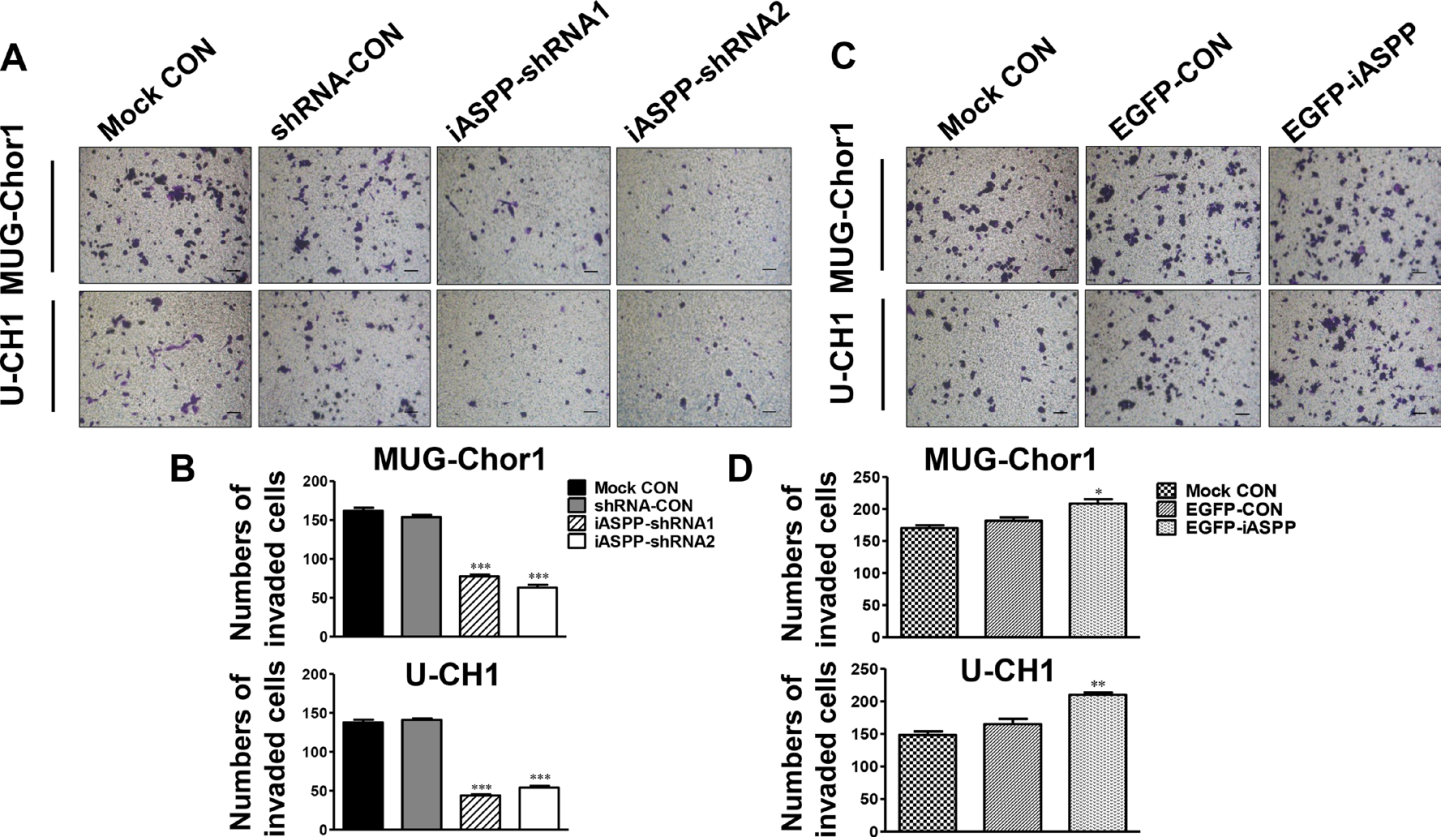
Effect of iASPP knockdown and overexpression on cells invasion (**A**) Pictures of transwell invasion assays in MUG-Chor1 and U-CH1 cells after the iASPP knockdown with iASPP-shRNAs. (**B**) The number of both cells that invaded the substratum of the membrane per view under a 100× magnification after the iASPP knockdown with iASPP-shRNAs. (**C**) Pictures of transwell invasion assays in MUG-Chor1 and U-CH1 cells after the iASPP overexpression with EGFP-iASPP. (**D**) The number of both cells that invaded the substratum of the membrane per view under a 100× magnification after the iASPP overexpression with EGFP-iASPP. *n* = 3. Mean ± SEM. *p < 0.05, ^*^p < 0.01, ^**^*p < 0.001, iASPP-shRNA1 group or iASPP-shRNA2 group *vs*. shRNA-CON group, and EGFP-iASPP group *vs*. EGFP-CON group. CON: control. Bar scale = 100 μm.

Results of transwell invasion assays showed that the number of invading cells was significantly reduced with iASPP downregulation in both chordoma cell lines in comparison with the corresponding control group (Figure 5A). The corresponding fold change is about 0.50-fold in the iASPP-shRNA1 group (*p <* 0.001) and 0.41-fold in the iASPP-shRNA2 (*p <* 0.001) for MUG-Chor1; and 0.31-fold in the iASPP-shRNA1 group (*p* < 0.001) and 0.38-fold in the iASPP-shRNA2 (*p* < 0.001) for U-CH1 (Figure 5B). However, iASPP overexpression remarkably promoted the invasion of these chordoma cell lines (Figure 5C). The fold change is approximately 1.13-fold (*p <* 0.05) for MUG-Chor1 and 1.34-fold (*p <* 0.01) for U-CH1 (Figure 5D).

### Cells sensitivity to cisplatin is enhanced by iASPP knockdown but attenuated by iASPP overexpression

**Figure 6:**
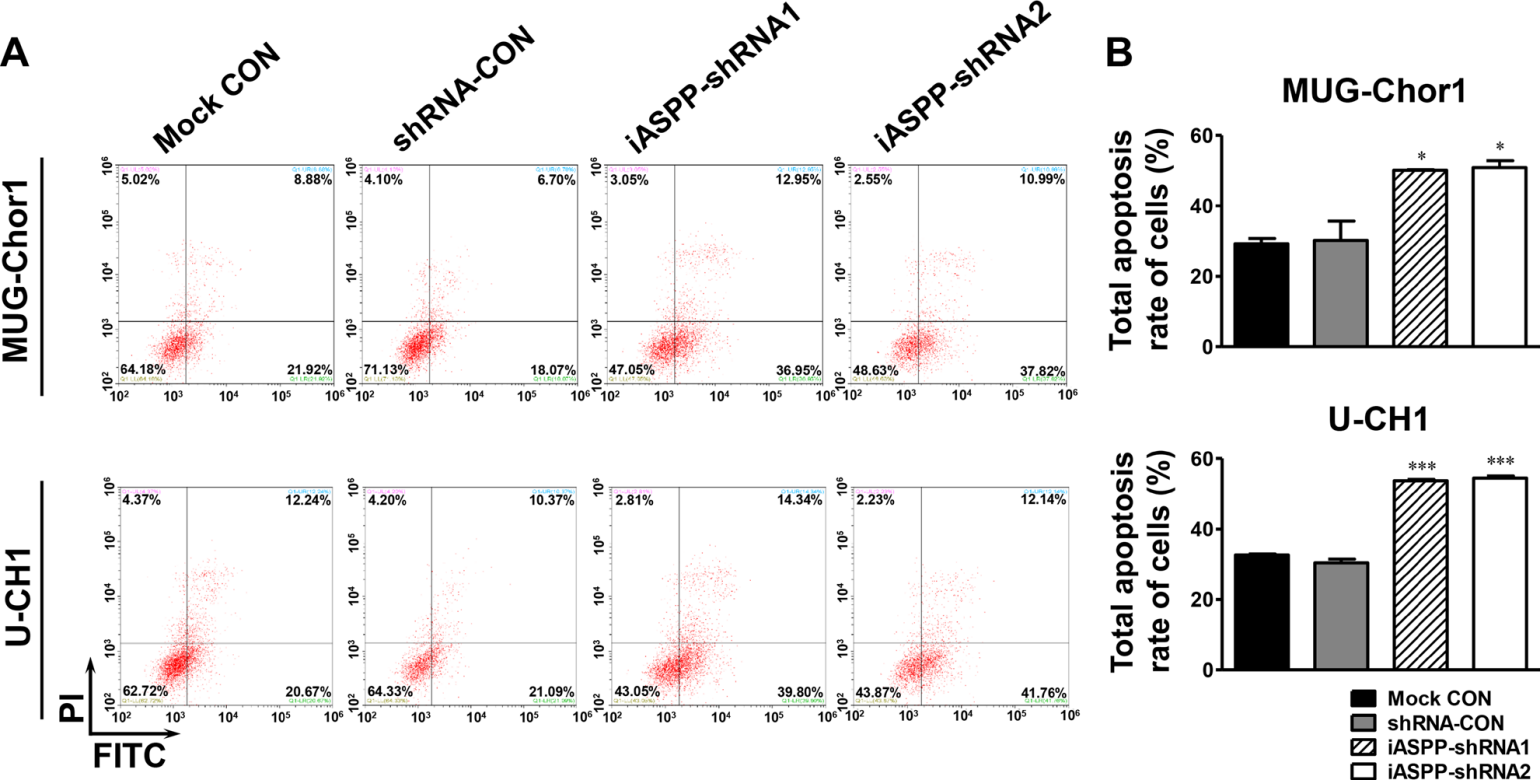
Effect of iASPP knockdown on cells apoptosis after cisplatin treatment (**A**) Pictures of cells apoptosis detected by flow cytometry in 20 μM cisplatin-treated MUG-Chor1 and U-CH1 cells after the iASPP knockdown with iASPP-shRNAs. (**B**) The results of statistical analysis of total apoptosis rate in 20 μM cisplatin-treated cells after the iASPP knockdown with iASPP-shRNAs. *n* = 3. Mean ± SEM. *p < 0.05, ^**^*p < 0.001, iASPP-shRNA1 group or iASPP-shRNA2 group *vs*. shRNA-CON group. CON: control.

**Figure 7:**
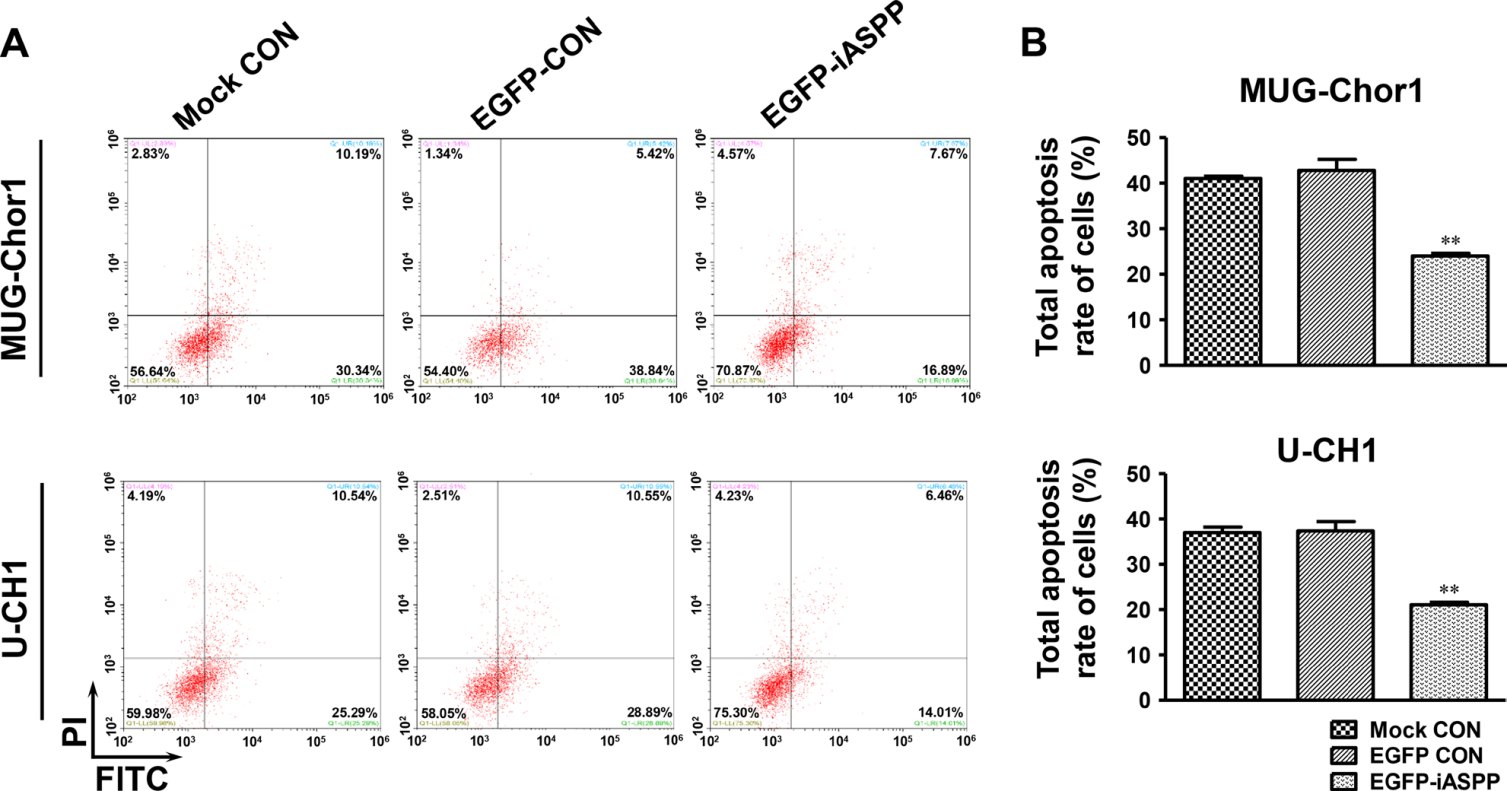
Effect of iASPP overexpression on cells apoptosis after cisplatin treatment (**A**) Pictures of cells apoptosis detected by flow cytometry in 20 μM cisplatin-treated MUG-Chor1 and U-CH1 cells after the iASPP overexpression with EGFP-iASPP. (**B**) The results of statistical analysis of total apoptosis rate in 20 μM cisplatin-treated cells after the iASPP overexpression with EGFP-iASPP. *n* = 3. Mean ± SEM. ^*^p < 0.01, EGFP-iASPP group *vs*. EGFP-CON group. CON: control.

After treatment with 20 μM cisplatin for 48 h, the apoptosis of both MUG-Chor1 and U-CH1 cells was obviously elevated upon iASPP silencing when compared to that in the controls (Figure 6A). For MUG-Chor1 cells, the apoptosis rate was about 50.06% in the iASPP-shRNA1 group (*p <* 0.05) and 50.84% in the iASPP-shRNA2 group (*p <* 0.05) *vs*. 30.22% in the shRNA control group (Figure 6B). For U-CH1 cells, the rate was about 53.74% in the iASPP-shRNA1 group (*p <* 0.001) and 54.50% in the iASPP-shRNA2 group (*p <* 0.001) *vs*. 30.42% in the shRNA control group (Figure 6B). In contrast, the apoptosis of both cells was reduced with iASPP overexpression when compared to that in the corresponding controls (Figure 7A). The apoptosis rate was approximately 23.97% in the EGFP-iASPP group *vs*. 42.78% in the EGFP-control group for MUG-Chor1 (*p <* 0.01), and 21.05% in the EGFP-iASPP group *vs*. 37.40% in the EGFP-control group for U-CH1 (*p <* 0.01) (Figure 7B).

### miRNA-124 down-regulates iASPP expression

**Figure 8:**
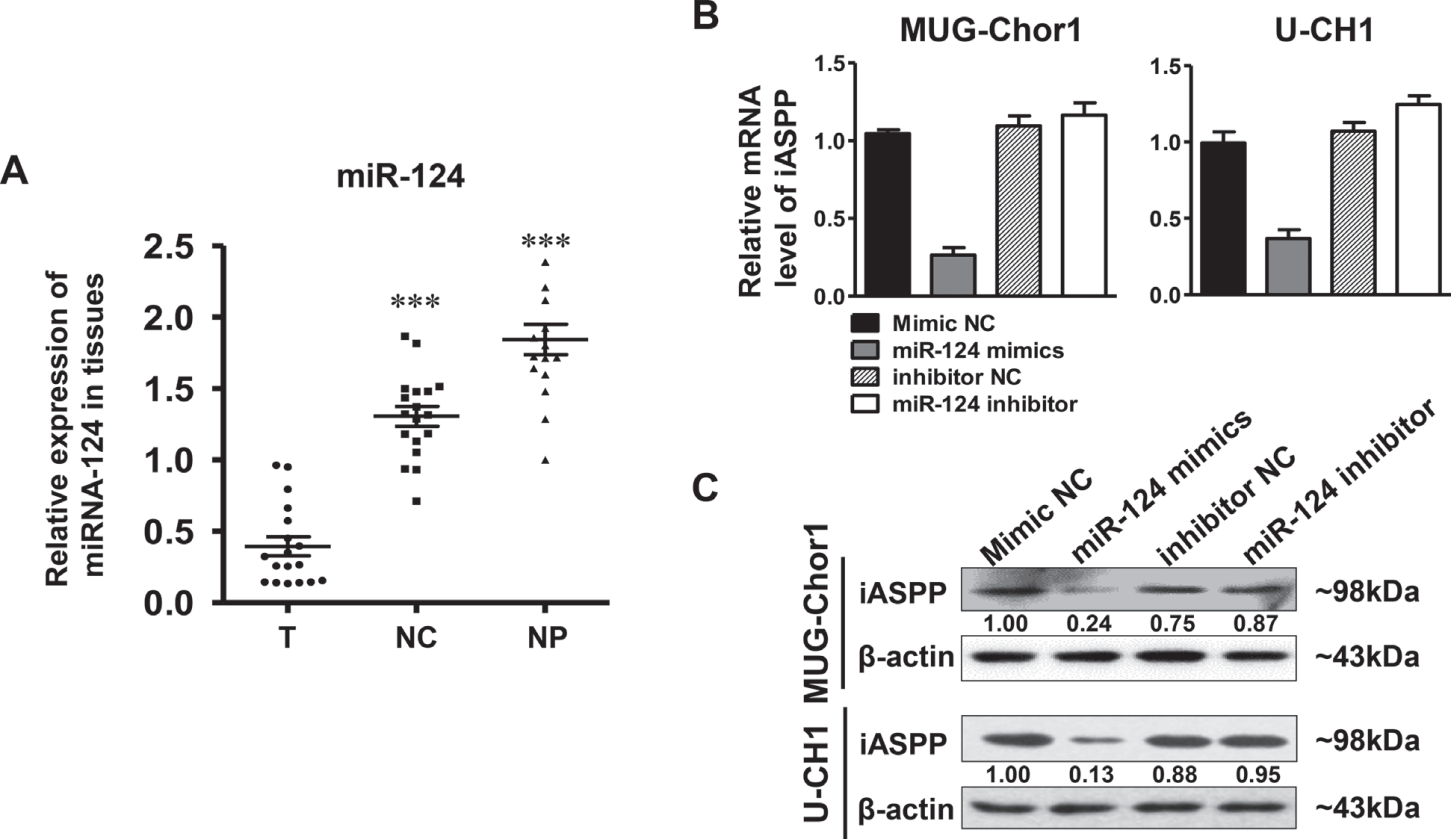
The expression of miRNA-124 in tissues and iASPP expression regulated by miRNA-124 (**A**) Relative expression of miRNA in tissues as detected by RT-qPCR. ^**^*p < 0.001 *vs*. tumor group. T: tumor group, NC: normal control tissues group, NP: nucleus pulposus tissues group. (**B**, **C**) Relative expression of iASPP in MUG-Chor1 and U-CH1 cells after the transfection of miRNA-124 mimics and inhibitor as detected by RT-qPCR (B) and WB (C). Numbers below picture C represent semi-quantitative analysis of each line by Quantity One software.

The expression of miRNA-124 in patients’ tissue samples was firstly determined by RT-qPCR. The results indicated that the level of miRNA-124 in the tumor group was significantly lower than that in the control groups that including distant normal tissues (*p* < 0.001) and NP tissues (*p* < 0.001) (Figure 8A). Subsequently, we further observed the regulation of miRNA-124 on iASPP expression in both MUG-Chor1 and U-CH1 cells. As shown in Figure 8B, miRNA-124 presented a negative regulation on iASPP expression at mRNA level in both cells after respectively transfecting cells with miRNA-124 mimics and inhibitor. iASPP was significantly down-regulated in cells transfected with miRNA-124 mimics but slightly up-regulated with miRNA-124 inhibitor. What's more, the same findings were also observed at protein level (Figure 8C). All these results suggested that the down-regulation of miRNA-124 existed in chordoma cells and its dysregulation inversely regulated the expression of iASPP.

### miRNA-124 inversely regulates the apoptosis function of iASPP

**Figure 9:**
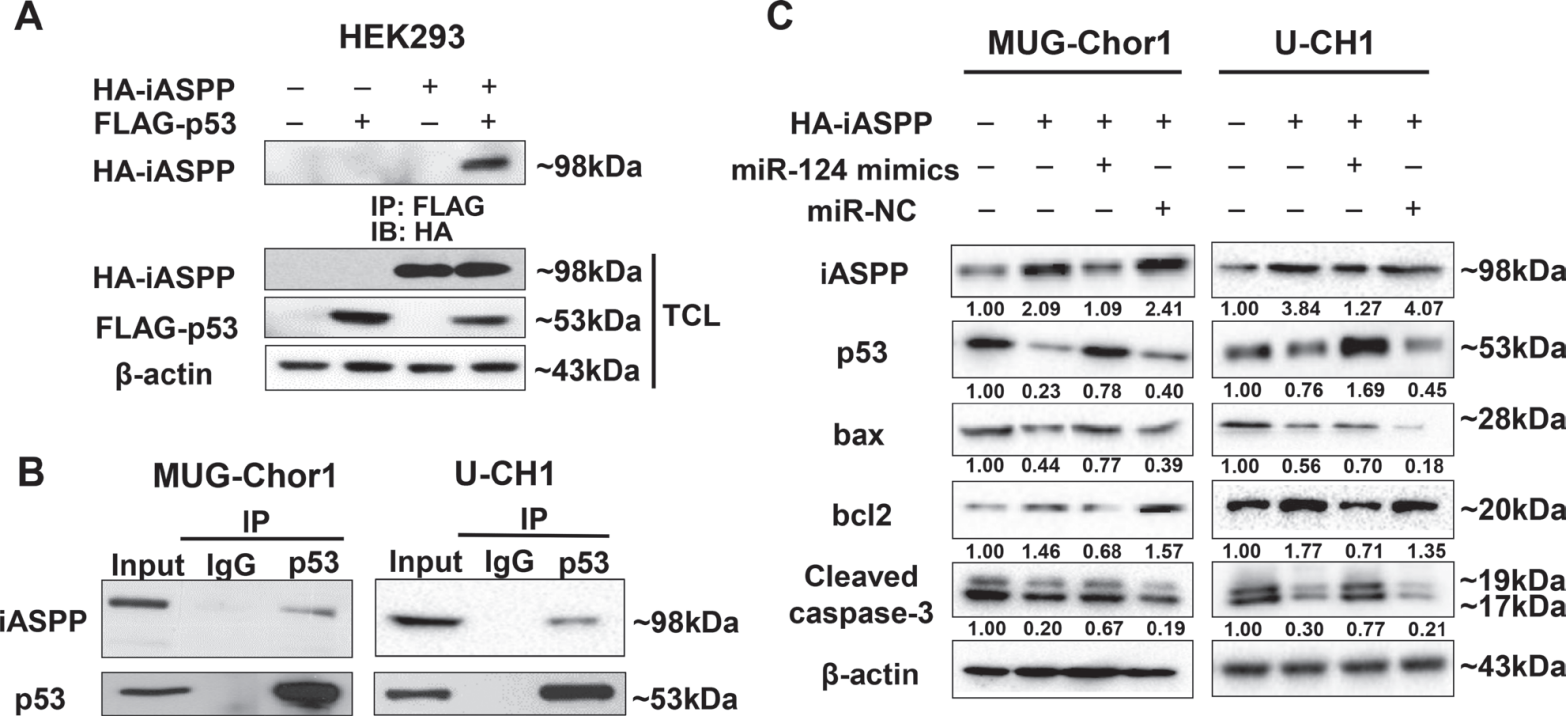
The interaction between iASPP and p53 detected by Co-Immunoprecipitation (Co-IP) assay and the regulation of miRNA-124 on apoptosis function of iASPP (**A**, **B**) The Co-IP detection of the interaction between iASPP and p53 in exogenous (A) and endogenous conditions (B). (**C**) Changes of apoptosis-related proteins based on the regulation of miRNA-124 on iASPP in MUG-Chor1 and U-CH1 cells. Numbers below picture C represent semi-quantitative analysis of each line by Quantity One software.

The co-immunoprecipitation for exogenous detection was performed in HEK293 cells that co-transfected with pCMV-FLAG-p53 and pCMV-HA-iASPP for 48 h. Figure 9A demonstrated that FLAG-p53 could interact with iASPP in exogenous detection. In order to observe the endogenous interaction between iASPP and p53, cell lysates of MUG-Chor1 and U-CH1 were immunoprecipitated with anti-p53 antibody and immunoblotted with anti-iASPP antibody. Figure 9B showed that p53 also interacted with iASPP in endogenous detection. All these data confirmed that the interaction between iASPP and p53 existed in chordoma cells.

By transfecting both chordoma cells with iASPP, iASPP + miRNA-124 mimics and iASPP + miRNA-NC, we also evaluated the potential effect of miRNA-124 targeted to iASPP on certain apoptosis-related proteins including p53, bax, bcl-2, and caspase-3. The results indicated that iASPP significantly inhibited p53 expression and up-regulated anti-apoptotic protein bcl-2, but obviously down-regulated pro-apoptotic proteins bax and caspase-3. However, the inhibition of iASPP by miRNA-124 mimics presented the reverse regulation effects on these proteins (Figure 9C).

## DISCUSSION

Chordoma mainly involves the axial skeleton with strong local invasiveness and a high recurrence rate. It is an intractable disease as even with complete resection, local recurrence is still common. Researchers have demonstrated that p53 dysfunction is one of the causes in tumorigenesis of chordoma; however, p53 mutation is not a common event in this disease [11, 38]. Evidence suggests that there are several other factors interrupt the normal function of p53. iASPP, the most evolutionarily conserved member in ASPP family, can inhibit apoptosis by specifically interfering with the transactivation of p53-family members to the promoters of apoptosis-related genes [14, 39]. Thus far, the abnormal expression of iASPP has been widely reported in many types of tumors and has a vital function in their occurrence and development [40, 41]. Cao et al. [23] demonstrated that the elevated iASPP in squamous cell cervical cancer correlated to patients’ poor prognosis and chemo/radiotherapy resistance. Kim et al. [42] showed that recurrent cases of oral cavity squamous cell carcinoma presented higher iASPP expression than primary cases, and these cases were associated with a lower survival rate. However, the function of iASPP in spinal chordoma and its clinical significance were previously unclear and explored in this study.

A total of 31 spinal chordomas were collected and tumor specimens were all confirmed pathologically to be conventional type by HE and IHC assays. For IHC assays, proteins including pan-cytokeratin, vimentin, epithelial membrane antigen (EMA), and S-100 were detected. These are considered as recognized biomarkers for the diagnosis of chordoma [43]. Brachyury, a novel tumor-specific biomarker for chordoma [44], was also assessed by IHC to confirm diagnosis. Subsequently, iASPP expression in chordoma tissues and two chordoma cell lines (MUG-Chor1 and U-CH1) were detected. Results demonstrated that iASPP predominantly expressed in the cytoplasm of chordoma cells and significantly overexpressed in chordoma tissues when compared to that in distant normal tissues and nucleus pulposus tissues. These results were consistent with the findings of iASPP expression in hepatocellular carcinoma and ovarian cancers that respectively reported by Lu et al. and Jiang et al. [18, 21]. IHC staining showed that iASPP was highly expressed in 67.7% (21/31) of chordoma samples, but only 11.1% (2/18) of distant normal tissues, and 26.7% (4/15) of nucleus pulposus tissues; in addition, these differences were statistically significant. Likewise, iASPP expression in two chordoma cell lines observed by RT-qPCR and WB was also obviously higher than the normal control of primary nucleus pulposus cells. These results indicate that iASPP might be a crucial diagnostic biomarker for chordoma.

Clinically, it has showed that certain factors such as tumor size and location, local invasion, and surgical types and margins closely correlated to local recurrence and prognosis in chordoma patients [6, 45, 46]. Meanwhile, Morris et al. [47] reported that elevated iASPP was associated with tumor invasive growth, metastatic disease, and cancer-related mortality. They observed that iASPP was enriched in highly metastatic prostate cancer cells compared to expression in primary cells, and that iASPP-overexpressing cells were mostly distributed at the invasive leading edge. Lu et al. [18] also observed that iASPP overexpression in hepatocellular carcinoma was closely associated with tumor recurrence and patient survival time. Consistently, our data suggested that elevated iASPP expression was significantly associated with tumor invasion, but not with patient age, gender, or tumor size and location. In addition, 76.2% (16/21) of patients presenting tumors with high iASPP expression developed recurrence, whereas recurrence occurred in only 20% (2/10) of patients having disease with low iASPP expression, and this difference was statistically significant. Furthermore, Kaplan-Meier survival plots and log-rank tests also suggested that the CDFS in patients with high iASPP expression was much shorter than in those with low iASPP expression. In addition, the cumulative survival rate of patients in the high iASPP expression group was notably lower than that in patients with the low expression group. Taken together, these results suggest that iASPP can serve as a novel prognostic factor and a predictor of tumor recurrence.

Many experimental studies have confirmed that the dysregulation of iASPP can notably disturbing the malignant biological behaviors of human cancers [14, 16, 17]. Chen et al. [16] suggested that the suppression of iASPP by employing shRNA technology led to an obvious inhibition of cellular proliferation in two non-small lung tumor cells harboring wild-type p53. Other studies also demonstrated that the proliferative and invasive properties of the pancreatic adenocarcinoma cells and the central nervous system lymphoma cells were remarkably weakened due to the downregulation of iASPP [34, 36]. Similar to these reports, the cellular proliferation and invasion of chordoma cells in this study significantly decreased with iASPP silence, but enhanced with iASPP overexpression. In fact, iASPP overexpression in human malignant cells mainly relates to their chemotherapy resistance [14]. Jiang et al. [21] observed that ovarian cancer cells with iASPP overexpression conferred resistance to paclitaxel by reducing mitotic catastrophe via activation of separase, whereas knockdown of iASPP enhanced paclitaxel-mediated mitotic catastrophe through inactivating separase. Jia et al. [22] reported that the p53-dependent apoptosis of hematopoietic cells with iASPP overexpression reduced in response to cell damage stimuli (etoposide/VP-16), and the mutation accumulation was more common in these cells. Furthermore, the downregulation of endogenous iASPP caused a significant increase of p53-mediated apoptosis in human malignances induced by chemotherapeutics [14, 48]. In this study, we also found that the apoptosis of chordoma cells notably increased with the knockdown of iASPP, but attenuated with iASPP overexpression. All these evidences imply that iASPP can be a potential therapeutic target for chordoma.

miRNAs are a class of non-coding, single-stranded RNAs with a small size (∼22 nucleotides in length), which negatively modulate gene expression at post-transcriptional level by mainly binding to the 3′-untranslated region (3′-UTR) of their target mRNAs [27]. Either the bioinformatics prediction of miRNA target based on sequence complementarity or the luciferase reporter assay used in previous studies both validated the binding and repressive effects of miRNA-124 on the 3′-UTR of iASPP [28]. The inversely regulation of miRNA-124 on iASPP expression has been confirmed to mediate the malignant progression including cellular proliferation, apoptosis and invasion in many tumor cells, such as glioblastoma, prostate cancer and colorectal cancer [30, 32, 33]. In this study, we observed that the level of miRNA-124 in chordoma tissues was significantly lower than the corresponding normal controls. What's more, there was a negative association between miRNA-124 and iASPP expression in chordoma cells validated by RT-qPCR and WB. These results suggested that iASPP overexpression was reversely regulated by miRNA-124, which was at a low level in chordoma.

The regulatory function on apoptosis by iASPP through p53 has been widely reported. Consistently, the results of co-immunoprecipitation assay confirmed that iASPP could interact with p53 in either exogenous or endogenous detection, which implied that the potential regulation of iASPP on the tumorigenesis of chordoma through p53. As a tumor suppressor, p53 exerts its function mainly by inducing cell cycle arrest or apoptosis, and p53-dependent apoptosis typically follow the mitochondrial pathway. Activation of cytoplasmic p53 is sufficient to increase the transcription of various pro-apoptotic Bcl-2 family members, such as Bax, Noxa, and Puma; in the mitochondrion, p53 directly induces Bax oligomerization and forms inhibitory complexes with the anti-apoptotic Bcl-xl and Bcl-2, which causes the permeabilization of the mitochondrial membrane and cytochrome c release [49]. These changes subsequently activate caspase cascades, and apoptotic effector caspase-3 is eventually activated to cause apoptosis [50]. The results showed that the level of p53 was significantly down-regulated by iASPP, followed by the up-regulation of anti-apoptotic protein bcl-2 and the down-regulation of pro-apoptotic proteins bax and caspase-3. However, the inhibition of iASPP by miRNA-124 mimics presented the reverse regulation effects on these proteins. These findings support that the overexpression of miRNA-124 can attenuate the inhibition of iASPP on p53, which potentially enhances the apoptosis of chordoma cells.

Besides, iASPP is considered as a substrate for caspases, which highlights its potential as a direct target that can be stimulated by apoptotic signals [51]. The signaling cascades of the Hedgehog/GLI-E2F1 axis or those downstream of hepatitis virus X protein-mediated NF-κB activation were also shown to modulate iASPP in tumor cells [18, 52]. These data indicate that the causes of iASPP overexpression in tumors might be multifarious, and thus need to be explored intensively. Although iASPP regulates the biological behaviors in tumor cells mostly through p53-dependent pathways, recent studies also report its regulation in a p53-independent manner [39, 41, 53]. These evidences suggest that the downstream regulatory mechanisms of iASPP are diverse and complicated. Therefore, it is necessary to explore these issues in the context of chordoma in future.

In conclusion, iASPP overexpression is associated with the clinical outcome in spinal chordoma and influences the cellular proliferation, invasion, and their sensitivity to cisplatin. It might serve as a novel prognostic and predictive biomarker as well as a therapeutic target for chordoma. In addition, further study is necessary to explore the deeply regulatory mechanisms of iASPP in the tumorigenesis and progression of chordoma.

## MATERIALS AND METHODS

### Patient data

A total of 31 chordoma patients (22 men and 9 women) were included in the study, with an average age of 55.0 years (range 20∼78 years). Lesions were located in the cervical vertebra in 19 patients and in the sacrococcygeal region in 12 patients. All patients received surgical treatment at the Department of Orthopedics, Peking University Third Hospital, from 2008 to 2015. Eighteen distant normal tissues obtained at least 3 cm from the surgical margins and 15 normal nucleus pulposus tissues derived from patients with lumbar disc herniation (LDH) or lumbar spinal stenosis (LSS) were collected as controls. The clinical information of chordoma patients was recorded in detail. The research was approved by the ethics committee of the Peking University Third Hospital (No. IRB00006761-2016048) and informed consent was obtained from all patients.

### IHC for iASPP

The EnVision two-step staining technique was performed in IHC to detect iASPP expression in tissue samples as described previously [23]. Briefly, the tissue sections were dewaxed with xylene and a gradient concentration of ethanol, which was followed by antigen heat retrieval. The primary antibody was a rabbit anti-iASPP polyclonal antibody (ab34898, 1:200, Abcam, Cambridge, MA, USA). Standard streptavidin-biotin-peroxidase complex method and a biotinylated anti-IgG secondary antibody were applied. Samples were visualized with 3, 3′-diaminobenzidine (DAB). In addition, tissue sections were incubated with phosphate-buffered saline buffer (PBS) to replace the primary antibody as a negative control (Supplementary Figure 3A). Sections of human breast carcinoma with known positivity were used as the positive control (Supplementary Figure 3B). All sections were observed under one optical microscope (Leica, Frankfurt, Germany) and scored independently by two experienced pathologists who were blinded to the patient information.

The results of IHC staining were analyzed by combining the percentage of positive cells with the staining intensity as similar to previous report [16]. The percentage of positively stained cells was scored as 0 (none), 1 (1∼25%), 2 (26∼50%), 3 (51∼75%), or 4 (76∼100%). The color intensity was scored as 0 (negative), 1 (weak), 2 (moderate), and 3 (strong). The total score was calculated by the product of the staining intensity and the percentage of positive cells. Thus, the range of the final score was 0–12. For statistical analyses, the expression of iASPP was divided into low expression (final score ≤ 6) and high expression groups (final score ≥ 8) according to the mean value of scores from all 31 data.

### Pathological diagnosis of chordoma samples

The pathological diagnosis of tumor samples was assessed by HE and IHC staining. The primary antibodies used in IHC staining included mouse anti-pan cytokeratin monoclonal antibody (ab7753, 1:250, Abcam, Cambridge, MA, USA), rabbit anti-vimentin monoclonal antibody (ab92547, 1:250, Abcam), rabbit anti-S100 polyclonal antibody (BS1318, 1:100, Bioworld Technology, Inc. St. Louis Park, MN, USA), mouse anti-EMA monoclonal antibody (ZM-0095, 1:100, ZSGB, Beijing, China), and rabbit anti-brachyury polyclonal antibody (sc-20109, 1:250, Santa Cruz Biotechnology, Santa Cruz, CA, USA).

### Follow-up

The imaging information for patients including the plain radiographs, computed tomography scans, and magnetic resonance images was collected. All patients were subjected to follow-up and the average follow-up period was 60.6 months (range 7∼98 months). During follow-up, local recurrence was identified in 18 cases and the mean recurrence time was 20.1 ± 14.8 months. At the end of the follow-up, 11 patients experienced cancer-related death. CDFS was defined as the time interval from resection of the first tumor to recurrence [54].

### Cell lines and cell culture

Two human chordoma cell lines, MUG-Chor1 (CRL-3219) and U-CH1 (CRL-3217), purchased from the American Type Culture Collection (ATCC, Manassas, VA, USA) were not passaged for longer than 6 months during the study. They were cultured in Dulbecco's modified Eagle's medium (DMEM) and RPMI medium with a volume ratio of 4:1 (HyClone, Logan, UT, USA) which supplemented with 2 mM L-glutamine (Gibco, Grand Island, NY, USA), 10% fetal bovine serum (FBS) (HyClone), 100 U/mL penicillin, and 100 μg/mL streptomycin (Gibco). Cell culture flasks or dishes were pre-coated with rat tail collage type I (Corning Lasertron, Bedford, MA, USA). Human Prostatic Cancer (PC3) cells were cultured in DMEM medium supplemented with the components as similar to chordoma cells but without L-glutamine as an iASPP positive control. Human Emborynic Kidney (HEK293) cells were cultured as same as the culture condition of PC3 cells. All cells were maintained in an incubator containing 5% CO_2_ at 37°C.

### Nucleus pulposus (NP) cell isolation and culture

The primary cell culture method of NP was similar to others [55]. Briefly, human intervertebral disc NP tissues derived from the patients with LDH or LSS were rinsed and minced into small fragments of about 1 mm^3^. Then, the tissues were isolated by digestion with 0.25% trypsin (Invitrogen, Carlsbad, CA, USA) for about 20 min and 0.1% type II collagenase (Invitrogen) for 3∼4 h at 37°C. The digested mixture was collected and centrifuged at 1,500 rpm for 10 min. After carefully removed the supernatant, the remnant was re-suspended with DMEM-F12 culture medium and plated in the flasks. When the primary cells were firmly attached, the media were changed every 3 days.

### Lentivirus packaging and infection

For knockdown of the endogenous iASPP gene (NM_001142502), shRNA targeting iASPP was cloned into the pLKD-CMV-U6 lentivirus vector (Obio Technology, Shanghai, China). Two effective shRNA target sequences were used as shRNA1 (5′-GCCTCAAAGGAGTAAAGTC-3′) and shRNA2 (5′-ACTACTCTATCGTGGATTT-3′), and a scrambled plasmid (5′-TTCTCCGAACGTGTCACGT-3′) was used as a negative control. The lentivirus expressing iASPP-shRNA1, iASPP-shRNA2, and the control sequence named as pLKD-iASPP-shRNA1, pLKD-iASPP-shRNA2, and pLKD-shRNA-Control, respectively. Similarly, the full length CDS of the *PPP1R13L* gene (encoding iASPP) was cloned into the pLenti-EF1a-EGFP-P2A-Puro-CMV-MCS-3Flag lentivirus vector, and the empty plasmid was used as a control. The lentivirus overexpressing iASPP and the corresponding control lentivirus named as pLenti-EGFP-iASPP and pLenti-EGFP-Control, respectively.

### RT-qPCR assay

Total RNA was extracted using TRIzol reagent (15596018, Invitrogen) and the cDNA of mRNA was synthesized from 1 μg of total RNA with M-MLV (M170A, Promega, USA). RT-qPCR was performed using a commercial SYBR Green RT-PCR Kit (QPK-201, Takara, Otsu, Japan). The reverse transcription and detection of miRNA was performed using Bulge-Loop^TM^ miRNA qRT-PCR Starter Kit (C10211, RiboBio Co., Guangzhou, China) and Bulge-Loop^TM^ hsa-miR-124-5p qRT-PCR Primer Set (miRQ0004591-1-2, RiboBio Co., Guangzhou, China). The detetion of U6 snRNA was used as internal reference (MQP-0202, RiboBio Co., Guangzhou, China). The amplification parameters in this assay as follows: 1 cycle at 95°C for 10 min, 40 cycles of 95°C 15 s and 60°C 1 min. The specific primers of *PPP1R13L* and GAPDH were constructed by Sangon (Sangon Biotech, Shanghai, China) and their detailed sequences as follows: *PPP1R13L* (forward: 5′-CAGACAGCGAGCTATGAACG-3′, reverse: 5′-GTGGCGCTAGTGAGGTTGTC-3′); GAPDH (forward: 5′-GGTGGTCTCCTCTGACTTCAACA-3′, reverse: 5′-GTTGCTGTAGCCAAATTCGTTGC-3′). The mRNA and miRNA expression was respectively normalized to the expression of GAPDH and U6, and was expressed as 2^−ΔΔCt^ after normalization. All samples were assayed in triplicate.

### WB assay

Total protein was extracted using RIPA lysis buffer (Applygen Technologies Inc., Beijing, China). Protein concentration was determined using the BCA method and sample preparation was performed as described previously [56]. Equal amounts of protein were separated using an SDS-PAGE gel (10%) and then transferred to a nitrocellulose membrane. Membranes were blocked with 5% BSA for 1 h and incubated with indicated primary antibodies as follows: anti-iASPP rabbit polyclonal antibody (ab34898, 1:1000, Abcam, Cambridge, MA, USA), anti-p53 rabbit monoclonal antibody (2527, 1:1000, Cell signaling technology, CST, Danvers, MA, USA), anti-bax rabbit polyclonal antibody (2772, 1:1000, CST), anti-bcl-2 rabbit polyclonal antibody (2872, 1:1000, CST), anti-caspase-3 rabbit polyclonal antibody (9662, 1:1000, CST), anti-FLAG rabbit polyclonal antibody (F7425, 1:2000, Sigma-Aldrich, St. Louis, MO, USA), and anti-HA TAG mouse monoclonal antibody (SAB1305536, 1:1000, Sigma-Aldrich). Anti-β-actin mouse monoclonal antibody (CW0096M, 1:3000, CWBIO, Beijing, China) was used as the internal control. IRDye 800CW-conjugated goat (polyclonal) anti-rabbit/mouse IgG antibody (1:10000, LI-COR^®^ Biosciences, Lincoln, NE, USA) was used as the secondary antibody. The bands were visualized using an Odyssey CLx infrared imaging system (LI-COR^®^ Biosciences) and its relative gray value was measured using by Quantity One software (Bio-Rad, Hercules, CA, USA).

### Co-Immunoprecipitation (Co-IP) assay

Plasmids encoding iASPP and p53 were constructed by digested PCR products and linked into the pCMV-HA (PT3283-5, Clontech, Takara Bio, USA) and pFLAG-CMV2 vectors (E7398, Sigma-Aldrich). HEK293 cells were inoculated into 10-cm dishes at a density of 2.0 × 10^7^ and then transfected with 8.0 μg pCMV-iASPP-HA plasmid combined with 7.0 μg pFLAG-p53 plasmid together at 80% confluence using lipofectamine 2000 reagent (Invitrogen) according to the protocol. Cells transfected with single plasmid or empty control plasmid was used as controls. At 48 h post-transfection, cells were collected for protein extraction with lysis buffer (100 mM NaCl, 2.5 mM MgCl_2_, 0.1mM EDTA and 0.01% Triton X-100, pH 8.0). An aliquot (1%) of lysis was isolated for WB analysis to detect the expression of iASPP and p53. Then, the remaining supernatants were incubated with FLAG-M2 beads (A2220, Sigma-Aldrich) at for 12 h at 4°C. Beads were washed three times with lysis buffer. The co-precipitated proteins were eluted and analyzed by WB with anti-HA monoclonal antibody.

To explore the endogenous interaction between iASPP and p53, MUG-Chor1 and U-CH1 cells were collected and lysis for protein extraction. The supernatant of cell lysates were collected and dithiobis (succinimidyl propionate) (DSP) (22585, Thermo Fisher Scientific, Waltham, MA, USA) was added into supernatant with 1 mg/mL for 2 h at 4°C. An aliquot (1%) of lysis was isolated for WB analysis to detect the expression of iASPP and p53. Then, the supernatant was immunoprecipitated overnight at 4°C with anti-p53 monoclonal antibody. The resultant Mab-protein complexes were immunoprecipitated with protein A/G agarose (sc-500775, Santa Cruz Biotechnology) for 2 h at 4°C. Then the agarose were washed three times with wash buffer (50 mM NaCl, 2.5 mM MgCl_2_, 0.1 mM EDTA and 0.01% Triton X-100, pH 8.0). The precipitates were then detected by WB with anti-iASPP antibody.

### Oligonucleotide transfection

MUG-Chor1 and U-CH1 cells were inoculated into 6-well plates at a density of 5.0 × 10^5^ per well and were transiently transfected with miRNA-124 mimics (miR10004591-1-5), miRNA-124 inhibitor (miR20004591-1-5), and relevant controls including micrON™ mimic Negative Control (miR01101-1-5) and micrOFF™ inhibitor Negative Control (miR02101-1-5) that purchased from RiboBio Co., Guangzhou, China. Lipofectamine RNAi MAX (Invitrogen) was used in transfection when cells reached 50% confluence. At 72 h post-transfection, cells were collected for the extraction of RNA and proteins, respectively. The effect of miRNA-124 on iASPP expression was determined by RT-qPCR and WB.

### CCK-8 cell proliferation assay

Cells were seeded in 96-well dishes at a final density of 5 × 10^3^ (MUG-Chor1) or 8 × 10^3^ (U-CH1) cells per well and incubated for 0, 4, and 7 d. At each time point, CCK-8 reagent (Dojindo Laboratories, Kumamoto, Japan) was added and incubated for 3 h according to the manufacturer's protocol. The mean absorbance at 450 nm from six wells was calculated. The experiment was repeated three times.

### Colony formation assay

Cells were seeded into 12-well plates (300 cells per well) and incubated for 2 weeks, and then fixed with paraformaldehyde and stained with crystal violet. The number of colonies in each well was counted under an inverted microscope (Leica, Frankfurt, Germany) and only more than 50 cells could be regarded as one colony. Three replicate wells were used for each condition, and the experiment was repeated three times.

### Annexin V-FITC/PI apoptosis assay

The apoptosis of cells that treated with 20 μM of cisplatin for 48 h was determined by flow cytometry using an Annexin V-FITC/PI apoptosis detection kit (BioVision Co, Milpitas, CA, USA). Briefly, 1 × 10^6^ cells were re-suspended in 300 μL of 1× binding buffer. Then, 5 μL of annexin V-FITC and 5 μL of PI reagent were added and samples were incubated for 10 min in the dark. The intensity analysis of fluorescence-activated cells was performed by flow cytometry (BD Biosciences, San Jose, CA, USA). All results were repeated for three times.

### Transwell invasion assay

A total of 5 × 10^3^ cells, re-suspended in 50 μL of serum-free medium, was added to the upper chamber, which was pre-coated with Matrigel (BD Biosciences, Franklin Lakes, NJ, USA) for the invasion assay. The lower chamber was filled with 600 μL of culture medium containing 10% FBS. After incubation for 36 h at 37°C, invading cells, on the lower surface of the filter membrane, were fixed by paraformaldehyde and stained with crystal violet. Under an inverted microscope, five random fields were selected to count under high magnification (200 ×). Each experiment was repeated three times.

### Statistical analysis

Data were analyzed using IBM SPSS statistics 20.0 software. A Pearson's chi-squared test was used to explore the association between iASPP expression and clinical data from patients. The Kaplan-Meier survival curve and log-rank test were applied to evaluate the correlation between iASPP expression and CDFS and cumulative survival rate in chordoma patients by univariate analysis. For comparison with the control group, a one-way analysis of variance (ANOVA) with a post hoc Dunnett analysis was performed. On statistical analysis, the data were analyzed based on the mean ± standard deviation (SD) and their presentations in figures were the mean ± standard error of the mean (SEM). The statistical significance was defined as a value of *p* < 0.05 based on two-tailed tests.

## SUPPLEMENTARY FIGURES



## Abbreviations

ASPP: apoptosis stimulating protein of p53
NF-κB: nuclear factor kappa B
RAI: Rel-associated inhibitor
NP: nucleus pulposus
CDS: coding domain sequence
HE: hematoxylin and eosin
IHC: immunohistochemistry
RT-qPCR: reverse transcription quantitative polymerase chain reaction
WB: Western blotting
CDFS: continuous disease-free survival time
EMA: epithelial membrane antigen
DAB: 3,3′-diaminobenzidine
PBS: phosphate-buffered saline buffer
ATCC: American Type Culture Collection
DMEM: Dulbecco's modified Eagle's medium
FBS: fetal bovine serum
Co-IP: Co-Immunoprecipitation
SEM: standard error of the mean
ANOVA: one-way analysis of variance
