# Correction: iASPP overexpression is associated with clinical outcome in spinal chordoma and influences cellular proliferation, invasion, and sensitivity to cisplatin *in vitro*

**DOI:** 10.18632/oncotarget.28395

**Published:** 2023-06-21

**Authors:** Yunlong Ma, Bin Zhu, Xiaoguang Liu, Zhongjun Liu, Liang Jiang, Feng Wei, Miao Yu, Fengliang Wu, Hua Zhou, Nanfang Xu, Xiao Liu, Lei Yong, Yongqiang Wang, Peng Wang, Chen Liang, Guanping He

**Affiliations:** ^1^Department of Orthopedics, Peking University Third Hospital, Beijing 100191, China; ^2^The Center for Pain Medicine, Peking University Third Hospital, Beijing 100191, China; ^*^These authors contributed equally to this work

**This article has been corrected:** In Figure 3A, the WB images for proteins iASPP and b-actin expression in UCH-1 cells have been duplicated and flipped. In addition, in Figure 5A, which shows the data of transwell invasion assays in MUG-Chor1 and U-CH1 cells after the iASPP knockdown, the ‘shRNA-CON’ images for the MUG-Chor1 and U-CH1 cells accidentally overlap. The corrected Figures 3 and 5, produced using the original data, are shown below. The authors declare that these corrections do not change the results or conclusions of this paper.

Original article: Oncotarget. 2017; 8:68365–68380. 68365-68380. https://doi.org/10.18632/oncotarget.20190

**Figure 3:**
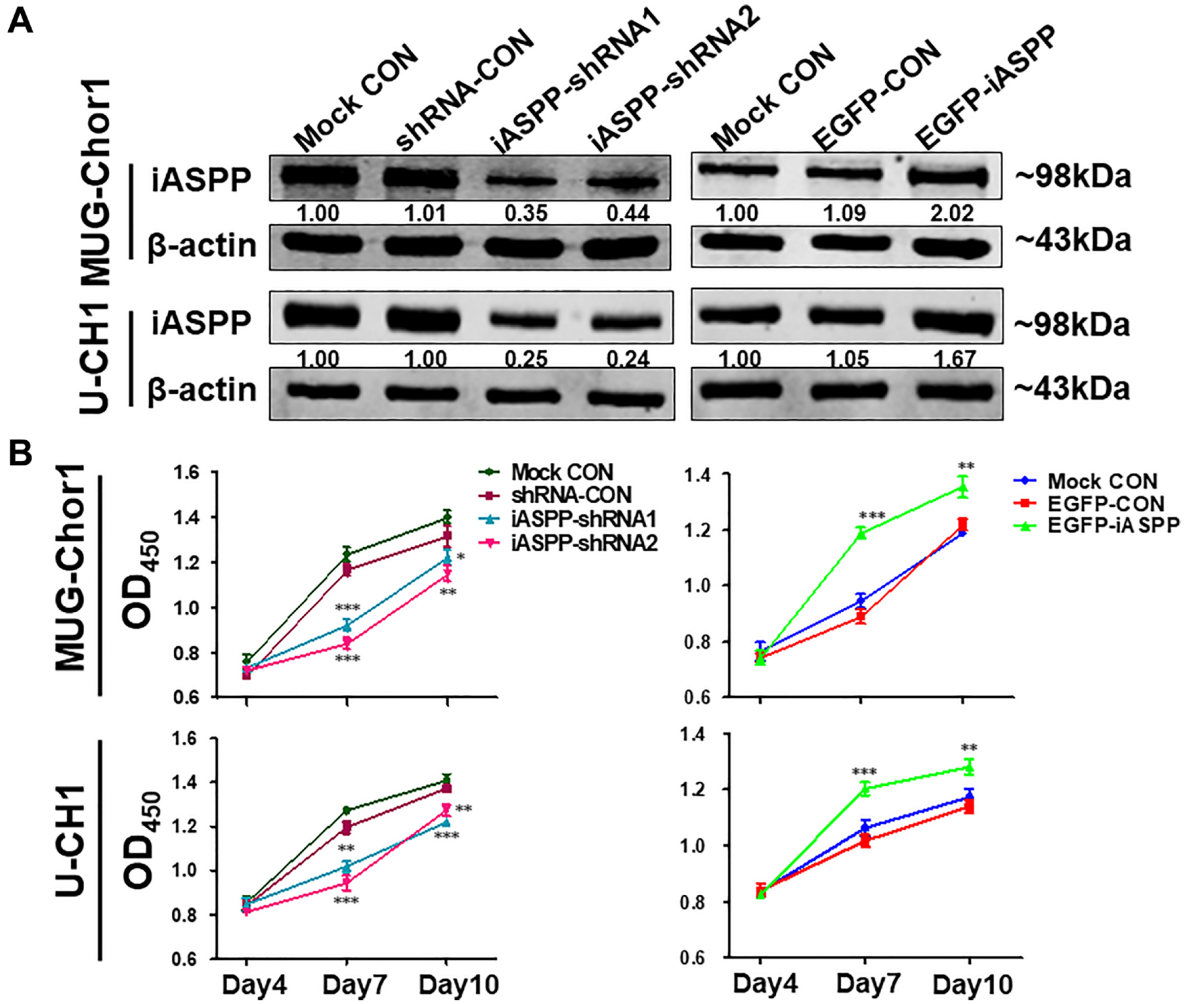
The extrinsic knockdown and overexpression of iASPP and their effects on cells proliferation based on CCK-8 assays. (**A**) The extrinsic knockdown and overexpression of iASPP in MUG-Chor1 and U-CH1 cells as identified by WB. Numbers below pictures represent semi-quantitative analysis of each line by Quantity One software. (**B**) Proliferation of both cells with iASPP knockdown and overexpression. *n* = 3. Mean ± SEM. ^*^ *p* < 0.05, ^**^ *p* < 0.01, ^***^ *p* < 0.001, iASPP-shRNA1 group or iASPP-shRNA2 group vs. shRNA-CON group, and EGFP-iASPP group vs. EGFP-CON group. CON: control.

**Figure 5:**
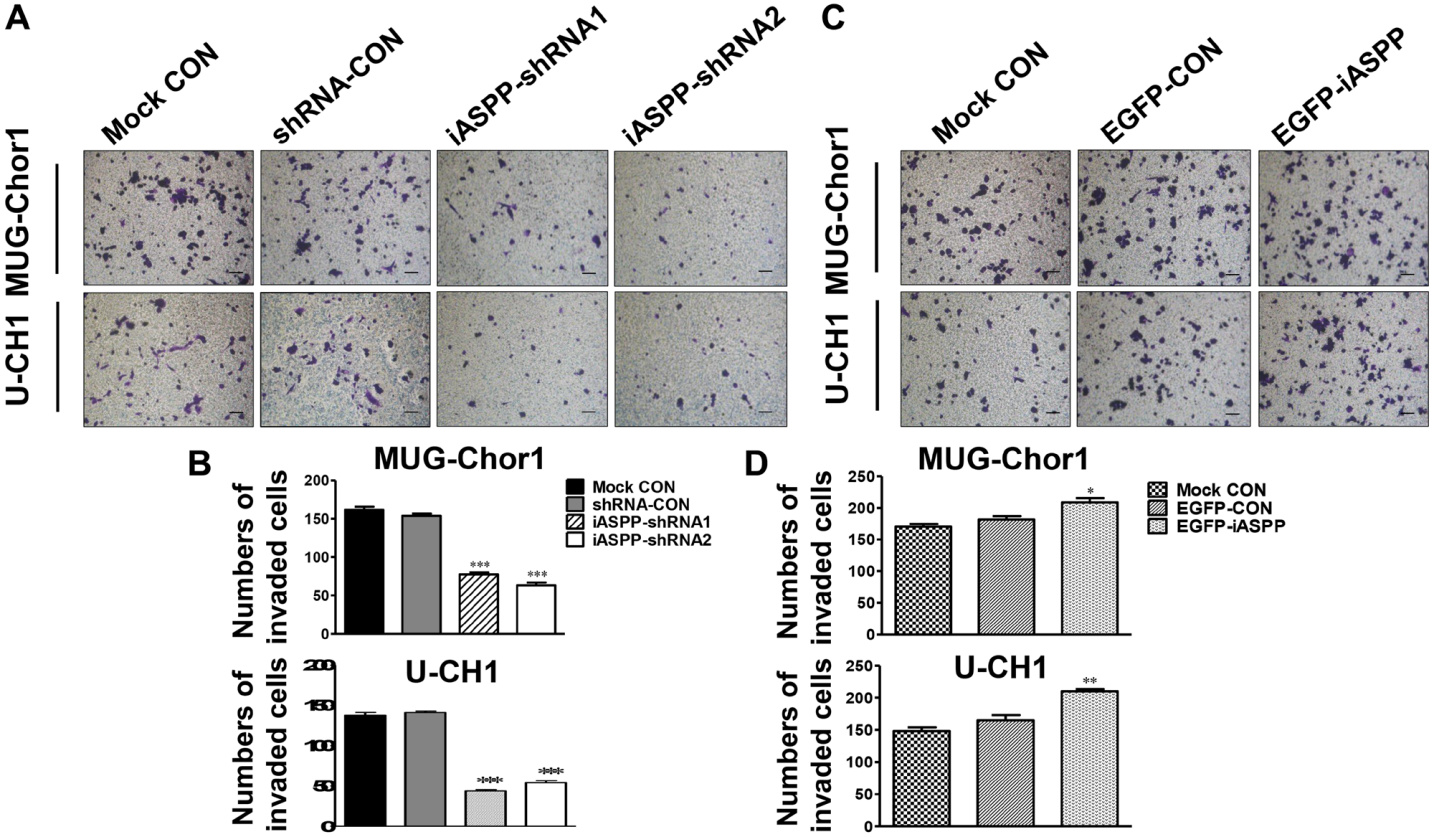
Effect of iASPP knockdown and overexpression on cells invasion. (**A**) Pictures of transwell invasion assays in MUG-Chor1 and U-CH1 cells after the iASPP knockdown with iASPP-shRNAs. (**B**) The number of both cells that invaded the substratum of the membrane per view under a 100× magnification after the iASPP knockdown with iASPP-shRNAs. (**C**) Pictures of transwell invasion assays in MUG-Chor1 and U-CH1 cells after the iASPP overexpression with EGFP-iASPP. (**D**) The number of both cells that invaded the substratum of the membrane per view under a 100× magnification after the iASPP overexpression with EGFP-iASPP. *n* = 3. Mean ± SEM. ^*^ *p* < 0.05, ^**^ *p* < 0.01, ^***^ *p* < 0.001, iASPP-shRNA1 group or iASPP-shRNA2 group *vs.* shRNA-CON group, and EGFP-iASPP group *vs.* EGFP-CON group. CON: control. Bar scale = 100 μm.

