# Supplementary material for: iASPP overexpression is associated with clinical outcome in spinal chordoma and influences cellular proliferation, invasion, and sensitivity to cisplatin in vitro
Source: Oncotarget. 2017 Aug 11;8(40):68365–80. doi: 10.18632/oncotarget.20190 (PMC5620263; doi:10.18632/oncotarget.20190)
Supplement: Supplementary file 1 [file oncotarget-08-68365-s001.pdf]

iASPP overexpression is associated with clinical outcome in spinal chordoma and influences cellular proliferation, invasion, and sensitivity to cisplatin *in vitro*

SUPPLEMENTARY MATERIALS

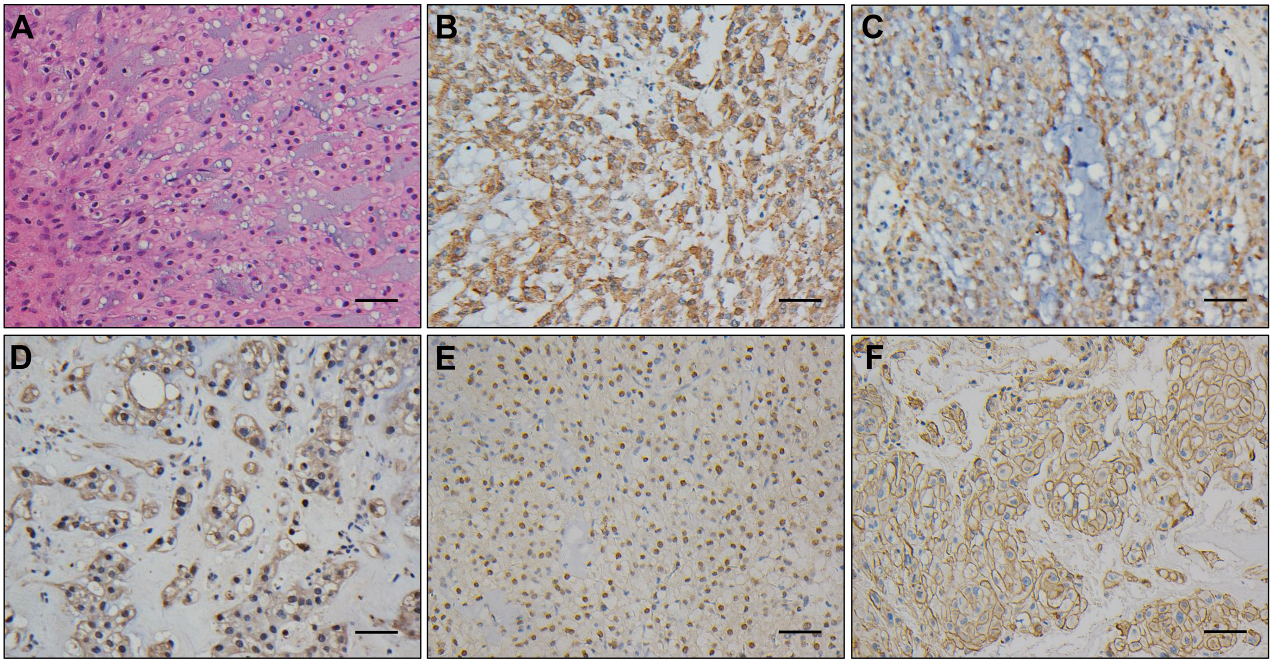

Supplementary Figure 1: Pathological diagnosis of chordoma tissues. (**A**) Hematoxylin eosin staining of chordoma tissues. (**B–F**) Immunohistochemistry staining with specific diagnostic biomarkers of chordoma, pan-cytokeratin (B), vimentin (C), S-100 (D), brachyury (E), and EMA (F). Bar scale = 50 μm.

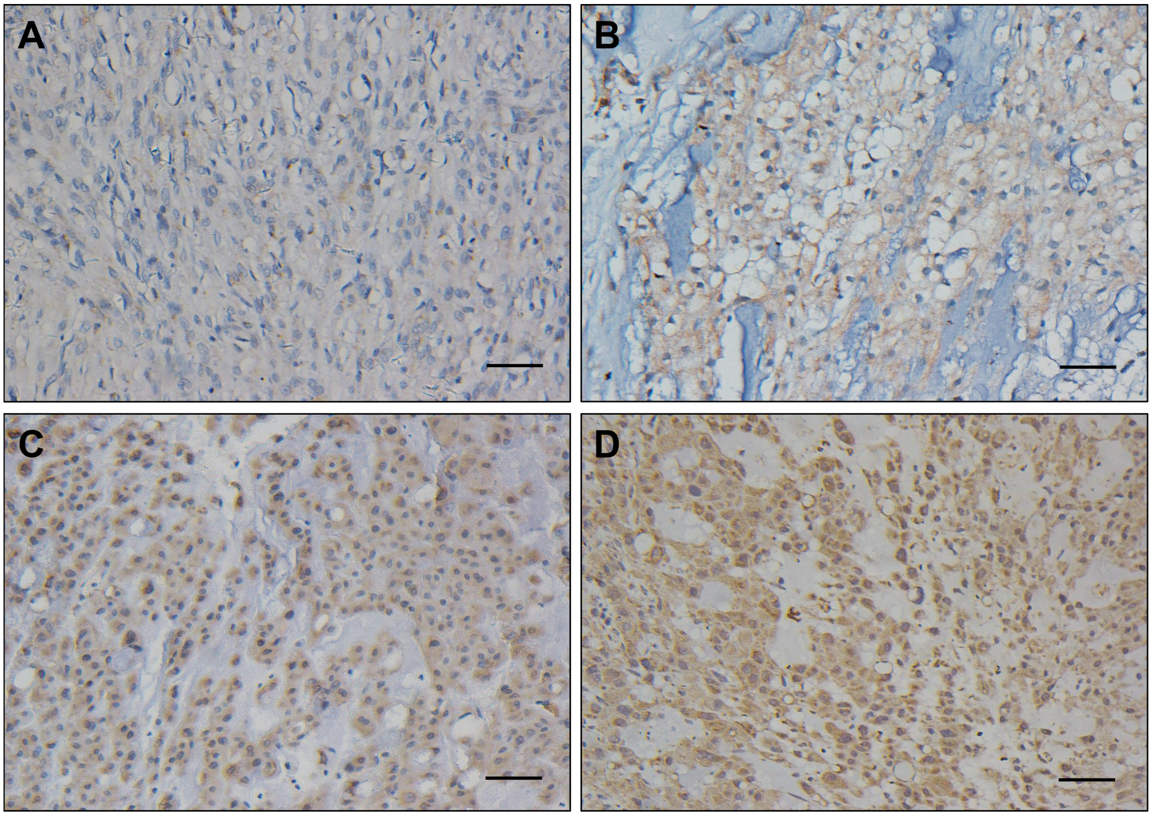

Supplementary Figure 2: Staining intensity of iASPP in chordoma tissues. Examples of (**A**) negative, (**B**) weak, (**C**) moderate, and (**D**) strong staining as assessed by two pathologists (described in Materials and Methods). Bar scale = 50 μm.

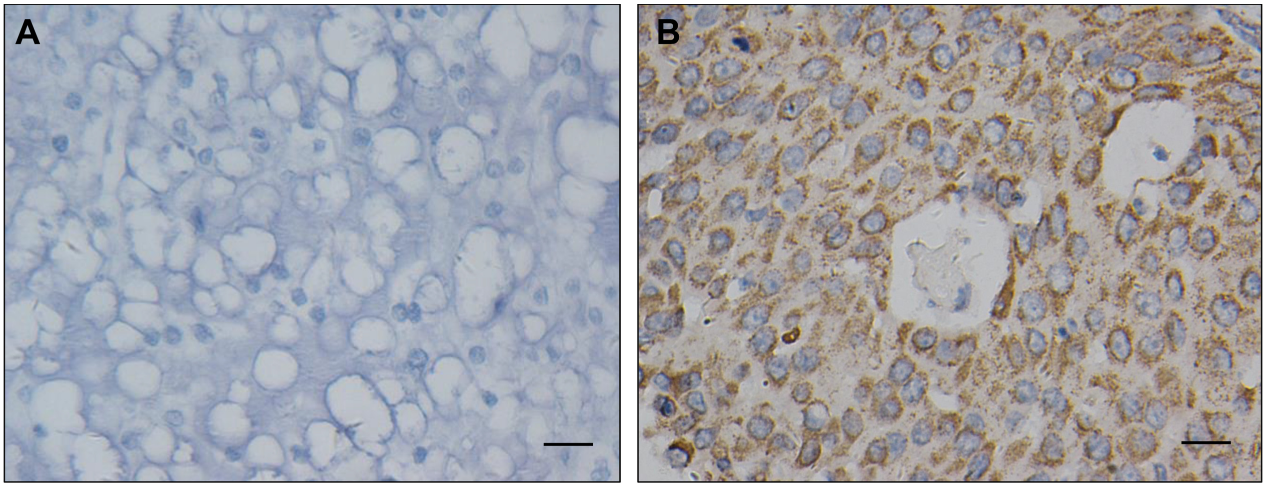

Supplementary Figure 3: The immumohistochemical pictures of negative control and positive control tissues. (**A**) Immumohistochemical staining of chordoma tissues incubated with phosphate-buffered saline buffer (PBS) to replace the primary antibody (negative control). (**B**) Immumohistochemical staining of human breast carcinoma with known iASPP positivity (positive control). Bar scale = 50 μm.
